# Paricalcitol Improves the Angiopoietin/Tie-2 and VEGF/VEGFR2 Signaling Pathways in Adriamycin-Induced Nephropathy

**DOI:** 10.3390/nu14245316

**Published:** 2022-12-14

**Authors:** Amanda Lima Deluque, Beatriz Magalhães Oliveira, Cláudia Silva Souza, Ana Lívia Dias Maciel, Heloísa Della Coletta Francescato, Cleonice Giovanini, Lucas Ferreira de Almeida, Francisco José Albuquerque de Paula, Roberto Silva Costa, José Antunes-Rodrigues, Terezila Machado Coimbra

**Affiliations:** 1 Laboratory of Renal Physiology, Department of Physiology, Ribeirao Preto Medical School, University of Sao Paulo, Ribeirao Preto 14040-900, SP, Brazil; 2 Laboratory of Endocrinology, Department of Internal Medicine, Ribeirao Preto Medical School, University of Sao Paulo, Ribeirao Preto 14015-010, SP, Brazil; 3 Laboratory of Renal Pathology, Division of Nephrology, Department of Internal Medicine, Ribeirao Preto Medical School, University of Sao Paulo, Ribeirao Preto 14015-010, SP, Brazil; 4 Laboratory of Neuroendocrinology, Department of Physiology, Ribeirao Preto Medical School, University of Sao Paulo, Ribeirao Preto 14040-900, SP, Brazil

**Keywords:** paricalcitol, endothelial toxicity, cell differentiation, angiopoietin-2, Tie-2 receptor, angiogenic factors, progressive kidney disease

## Abstract

Renal endothelial cell (EC) injury and microvascular dysfunction contribute to chronic kidney disease (CKD). In recent years, increasing evidence has suggested that EC undergoes an endothelial-to-mesenchymal transition (EndoMT), which might promote fibrosis. Adriamycin (ADR) induces glomerular endothelial dysfunction, which leads to progressive proteinuria in rodents. The activation of the vitamin D receptor (VDR) plays a crucial role in endothelial function modulation, cell differentiation, and suppression of the expression of fibrotic markers by regulating the production of nitric oxide (NO) by activating the endothelial NO synthase (eNOS) in the kidneys. This study aimed to evaluate the effect of paricalcitol treatment on renal endothelial toxicity in a model of CKD induced by ADR in rats and explore mechanisms involved in EC maintenance by eNOS/NO, angiopoietins (Angs)/endothelium cell-specific receptor tyrosine kinase (Tie-2, also known as TEK) and vascular endothelial growth factor (VEGF)-VEGF receptor 2 (VEGFR2) axis. The results show that paricalcitol attenuated the renal damage ADR-induced with antiproteinuric effects, glomerular and tubular structure, and function protection. Furthermore, activation of the VDR promoted the maintenance of the function and structure of glomerular, cortical, and external medullary endothelial cells by regulating NO production. In addition, it suppressed the expression of the mesenchymal markers in renal tissue through attenuation of (transforming growth factor-beta) TGF-β1/Smad2/3-dependent and downregulated of Ang-2/Tie-2 axis. It regulated the VEGF/VEGFR2 pathway, which was ADR-deregulated. These effects were associated with lower AT1 expression and VDR recovery to renal tissue after paricalcitol treatment. Our results showed a protective role of paricalcitol in the renal microvasculature that could be used as a target for treating the beginning of CKD.

## 1. Introduction

The progression of chronic kidney disease (CKD) to end-stage renal disease (ESRD) is related to the onset and evolution of interstitial renal fibrosis, regardless of the underlying cause. Interstitial renal fibrosis is characterized by the accumulation of fibroblasts and their activation into myofibroblasts, leading to an increase in extracellular matrix deposition [1]. Different types of cells are involved in kidney fibrosis, with fibroblasts as one of the primary mediators of this process. These fibroblasts may originate from the proliferation of resident fibroblasts, recruitment via bone marrow differentiation, epithelial-mesenchymal transition (EMT), and pericytes [2,3]. Additionally, new evidence has demonstrated the role of endothelial cells (EC) in this process [4]. EC may undergo an endothelial-to-mesenchymal transition (EndoMT), which can contribute to about 10% of the origin of these interstitial fibroblasts in response to many molecules, such as transforming growth factor-beta (TGF-β1) [2]. Furthermore, studies have shown a decrease in the endothelial surface layer (ESL) thickness, which triggers albuminuria in adriamycin-induced injury (ADR) [5]. Glomerular EC dysfunction initiates the development and progression of glomerulopathy. These findings indicate that EC alterations precede podocyte injury [6]. The ADR-induced nephropathy model in rodents is considered one of the most used to mimic focal segmental glomerulosclerosis (FSGS) and nephrotic syndrome (NS), characterized by renal dysfunction, glomerular fibrosis resulting in progressive albuminuria [7], and inflammation in the tubulointerstitial compartment due to the reabsorption of filtered proteins [8].

In healthy kidneys, the balance between pro- and anti-angiogenic factors is essential to renal vascular homeostasis. However, in CKD, this balance is interrupted [9,10], resulting in microvessel loss due to the anti-angiogenic environment, a process known as vascular rarefaction [11]. Angiopoietins (Ang-1 and Ang-2), a family of vascular growth factors, play a significant role in kidney vasculature homeostasis. The binding of Ang-1 to endothelium cell-specific receptor tyrosine kinase (Tie-2, also known as TEK) presents anti-inflammatory and pro-angiogenic activities, whereas Ang-2 has opposite effects. Angs are related to renovascular growth in parallel with vascular endothelial growth factor (VEGF), with roles in endothelial differentiation and survival [12], associated with endothelial repair capacity, endothelial nitric oxide synthase (eNOS), and the availability of nitric oxide (NO) [5]. In the ADR model, downregulation of the eNOS phosphorylation contributes to endothelial dysfunction in the heart, resulting in vascular endothelial dysfunction [13]. Therefore, a better understanding of EC participation at the beginning of the pathogenesis of progressive kidney injury may contribute to developing new therapeutic approaches.

Some evidence has proposed a potential therapeutic role of vitamin D (vit. D) in CKD [14,15,16]. Paricalcitol (19-nor-1,25-hydroxyvitamin D2), an analog of calcitriol, and vit. D receptor activators (VDRA) can prevent parathyroid hormone (PTH) secretion and secondary hyperparathyroidism (SHPT) in CKD [17]. Many clinical studies have shown the effects of paricalcitol therapy to attenuate CKD, indicated by efficiency and safety in PTH levels and calcium-phosphorus regulation, and in the reduction in the proteinuria progression when associated with renin-angiotensin system (RAS) blocker in a non-SHPT CKD [18]. RAS activation in renal tissue can be associated with TGF-β1 expression. The VDR activation suppresses these effects, attenuating EndoMT by regulation of the angiotensin II (AngII) signaling through the AngII type I receptor (AT1) [19]. A previous study has reported that paricalcitol improves hypoxia-induced injury in pericytes on kidney fibrosis by the TGF-β1/small mothers against decapentaplegic (Smad) pathway and α-smooth-muscle-actin (α-SMA) [20]. VDR activation from paricalcitol ameliorates EC function in 5/6 nephrectomy, independent of PTH levels, and can improve aortic relaxation [21]. Martínez-Miguel et al. [22] have observed that EC treated with vit. D significantly increased NO production by eNOS bioactivity. Strategies to maintain the integrity of ECs with higher NO bioavailability can reduce or prevent kidney injury. In the current literature, little is known about the vit. D effect on endothelial markers, such as Tie-2, Ang-1, and Ang-2 in the ADR-induced nephropathy, even though it has the glomerular EC as a target. Anti-angiogenic factors deficiency, such as thrombospondin-1 and Ang-2, improves proteinuria and renal structure [23,24,25]. Furthermore, the protective effects of vit. D are supported by our previous work showing that the lack of vit. D during renal development generated glomerular and peritubular capillary rarefaction due to an increased expression of Ang-2 and decreased expression of the renal axes Ang-1/Tie-2, VEGF/VEGFR2, and eNOS/NO, which cause disturbances in EC differentiation in adulthood [11] while vit. D supplementation increases eNOS and VEGF in unilateral ureteral obstruction (UUO) [26]. Thus, the literature supports the link between vit. D and their effects on endothelial function, and a deficiency in serum vit. D levels has been associated with kidney endothelium dysfunction.

The present work aimed to evaluate the effect of paricalcitol treatment on renal endothelial toxicity in a model of CKD induced by ADR in rats and elucidate two potential mechanisms involved in EC maintenance: Angs/Tie-2 and VEGF/VEGFR2.

## 2. Materials and Methods

### 2.1. Animals and Experimental Design

The ethical principles were performed in all experiments for animal experimentation of the Brazilian College of Animal Experimentation, and the Animal Experimentation Committee of the University of São Paulo at Ribeirão Preto Medical School approved the study protocol (COBEA/CETEA/FMRP-USP, protocol no. 194/2017). The animals were housed in a controlled temperature (22 °C) environment and exposed to a 12 h light/12 h dark cycle. They were provided with a chow diet and water ad libitum. Male Sprague-Dawley (180–200 g) were randomly selected for this study.

**Figure 1:**
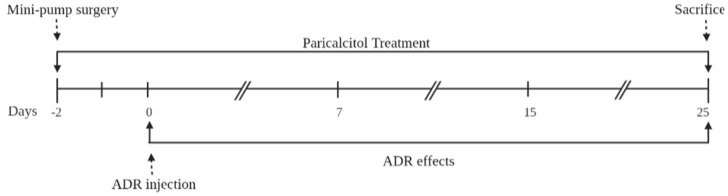
The schematic representation of study design. Male Sprague-Dawley rats underwent mini osmotic pump implanted surgery paricalcitol content two days before ADR administration and continued throughout the observation for 27 days. The animals were divided into 4 experimental groups: Control (*n* = 6): rats that received the vehicle; paricalcitol (*n* = 6): rats that received paricalcitol and i.v. of the vehicle; ADR (*n* = 7): rats that received i.v. of adriamycin, and ADR + paricalcitol (*n* = 7): rats that received paricalcitol and i.v. of the adriamycin.

Paricalcitol (6 ng/day, Zemplar^®^, Abbvie Laboratories, North Chicago, IL, USA) and vehicle (0.9% NaCl solution) were administered through a mini osmotic pump (Model 2004, Alzet, Cupertino, CA, USA) implanted in the back of the animal. This dose was selected according to the previously described [27]. Paricalcitol treatment started two days before adriamycin (ADR) administration and continued throughout the observation for 27 days, allowed by the mini osmotic pump model approach. In this case, 48 h after the procedure, the animals received an intravenous (i.v.) injection of ADR (Doxorubicin Hydrochloride/Fauldoxo^®^, Libbs, Brazil = 3.5 mg/kg) or vehicle through the tail vein as described previously [28] (Figure 1). The animals were divided into 4 experimental groups: Control (*n* = 6) being the rats that received the vehicle, paricalcitol (*n* = 6) being the rats that received paricalcitol and i.v. of the vehicle, ADR (*n* = 7) being the rats that received i.v. of the adriamycin, and ADR + paricalcitol (*n* = 7) being the rats that received paricalcitol and i.v. of the adriamycin.

### 2.2. Evaluation of Renal Function

The rats were housed in metabolic cages for 24-h urine sample collection on the 7th, 15th, and 25th days after ADR injection. These samples were stored at −20 °C until used for measuring progressive urinary albumin excretion (UAE) using the ELISA method with rat anti-albumin antibody (Bethyl Laboratories, Lab Research, Montgomery, TX, USA) and urinary creatinine using a commercial kit (Labtest Diagnostica, Lagoa Santa, Brazil). Furthermore, on the 25th day after ADR injection, we collected 24-h urine to measure urine volume, sodium (9180-electrolyte analyzer, Roche, Wien, Austria), creatinine (using a commercial kit Labtest Diagnostica, Lagoa Santa, Brazil), and NO levels. On the 26th day, the rats were weighed and then anesthetized using ketamine/xylazine (0.1 mL/100 g, Cristália, Itapira, Brazil). The blood samples (plasma and serum) were collected from the abdominal artery and stored at −70 °C. Plasma was used to analyze creatinine to calculate the glomerular filtration rate (GFR), sodium to perform fractional sodium excretion (FENa), NO levels, and calcium and phosphorus concentrations. Levels of 25 hydroxyvitamin D (25 OHD) and PTH were measured in serum samples. The urine samples were collected directly from the urinary bladder at the time of euthanasia, treated with 1 mM phenylmethylsulfonyl fluoride (PMSF; Sigma Chemical Company, St. Louis, MS, USA), and were stored at −70 °C until analysis for TGF-β1 levels. Kidneys were removed. One was fixed using methacarn solution for histological and immunohistochemical analyses, and the other was stored at −70 °C for ELISA and Western blot analyses.

### 2.3. Serum 25 Hydroxyvitamin D (25 OHD) Levels, Serum Parathyroid Hormone (PTH), Plasma Calcium (P_Ca_), and Phosphorus (P_P_) Levels Measurement

We assessed 25(OHD) with a direct competitive test based on the chemiluminescence principle (CLIA) (DiaSorin, Liaison^®^, Saluggia, Italy). This test was performed in the clinical analysis laboratories at the School of Medicine of Ribeirao Preto Hospital and Clinics, which participates in national and international quality assurance certification. The PTH levels were measured using the enzyme-linked immunosorbent assay (ELISA) method with intact rat anti-PTH (Quidel Corporation, San Diego, CA, USA), and plasma calcium and phosphorus levels were measured using commercial kits (Labtest Diagnostica, Lagoa Santa, Brazil).

### 2.4. NO in Urine, Plasma, and Renal Tissue Measurement

Samples of urine from the 25th day of the experiment, plasma, and renal tissue samples, were mixed/homogenized with 0.1 N acetic acid (3:1), centrifuged at 10,000× *g* for 5 min, and aliquoted. Only the kidney tissues were deproteinized with 95% ethanol (4 °C) (1:2) and centrifuged (4000× *g* for 5 min) again. The supernatants of urine, plasma, and kidney tissue were subjected to an analysis of NO content using the NO/ozone technique described previously with a Sievers analyzer (Sievers 280 NOA, Frederick, CO, USA) [27]. As described in previous studies, the Bradford method was used to determine protein levels in renal tissue [27]. The median NO values are expressed in µM/µg of protein in the renal tissue and µg/mg of plasma or urine creatinine.

### 2.5. TGF-β1 Measurement

Samples of urine taken from the bladder and renal tissue were used to quantify the transforming growth factor (TGF)-β1 content by ELISA (Promega Corporation, Madison, WI, USA). The results were expressed as pg/mg of creatinine or pg/mg of protein. As described in previous studies, the Bradford method was used to determine protein levels in renal tissue [27].

### 2.6. Histological Analysis

Kidney tissues were embedded in paraffin, sliced into 4-μm-thick slices, stained with Masson’s Trichrome (MT), and visualized using a light microscope (AxioVision Rel. 4.3; Zeiss, Oberkochen, Germany). In this case, 20 consecutive 0.1 mm^2^ fields of the cortex and 20 consecutive 0.1 mm^2^ fields of the outer medullary compartment were evaluated. Cortical and medullary tubulointerstitial MT expression was quantified using the NIH Image J software 1.52A (Bethesda, MD, USA), and mean values per kidney were calculated. Images are taken and quantified at high magnification (400×). The results were expressed as a percentage of fibrosis in the cortex and medullary compartment.

### 2.7. Immunohistochemical Analysis

The kidney sections were deparaffinized and hydrated for immunohistochemical analysis. Non-specific antigen binding was blocked by incubation for 20 min with normal goat serum. The sections were then incubated with anti-aminopeptidase P (JG12, BMS1104, 1:500, eBioScience, San Diego, CA, USA) for 60 min at room temperature, anti-vimentin (M0725, 1:50, Dako Corporation, Glostrup, Denmark), anti-α-smooth-muscle-actin (α-SMA [M0851, 1:50, Dako Corporation, Glostrup, Denmark]), anti-collagen I (Col I [AB755, 1/1200, Chemicon, Chicago, IL, USA]) and anti-desmin (M0760, 1/50, Dako Corporation, Carpinteria, CA, USA) antibodies overnight 4 °C. Avidin-biotin-peroxidase complex (Vector Laboratories, Newark, CA, USA) and DAB (3,3′-diaminobenzidine [Sigma Chemical Company, St. Louis, MO, USA]) were used for detection. The sections were then counterstained with methyl green, dehydrated, and mounted. Images are taken and quantified at high magnification (400×).

In this case, 30 consecutive 0.1 mm^2^ fields from the cortex and 20 consecutive 0.1 mm^2^ fields from the outer medullary compartment were evaluated for the JG12, α-SMA, vimentin, and Col I. Here, 30 cortical and 20 juxtamedullary glomeruli were evaluated for JG12, α-SMA, Col I, and desmin. Cortical and medullary tubulointerstitial changes were quantified using the NIH Image J software (Bethesda, MD, USA), and mean values per kidney were calculated. The results were expressed as a percentage of the positive cell in the glomerulus, cortex, and outer medulla.

### 2.8. Western Blot Analysis

The renal tissues were homogenized in a lysis buffer (50 mM Tris_HCl, pH 7.4; 150 mM NaCl; 1% Triton X-100; 0.1% SDS; 1 μg/mL aprotinin; 1 μg/mL leupeptin; 1 mM phenylmethylsulfonyl fluoride; 1 mM sodium orthovanadate, pH 10; 1 mM sodium pyrophosphate; 25 mM sodium fluoride; 0.001 M EDTA, pH 8) and centrifuged at 4 °C at 10,000 rpm. The proteins (30, 60 μg, or 90 μg) were separated by polyacrylamide gel electrophoresis, transferred to nitrocellulose membranes, incubated for one h in blocking buffer (TBS, 5% skim milk) or 3% BSA, washed in buffer (TBS, 0.1% Tween 20, pH 7.6) and then incubated with anti-phospho-endothelial nitric oxide synthase (p-eNOS, sc-12972, 1:200, Santa Cruz Biotechnology, Santa Cruz, CA, USA); anti-endothelial nitric oxide synthase (eNOS, sc-376751, 1:200, Santa Cruz Biotechnology, Santa Cruz, CA, USA); anti-α-smooth-muscle-actin (α-SMA, M0851, 1:300, Dako Corporation, Glostrup, CPH, Denmark); anti-vimentin (M0725, 1:1000, Dako Corporation, Glostrup, CPH, Denmark); anti-phospho-small mothers against decapentaplegic 2/3 (p-Smad2/3, 8828S, 1:500, Cell Signaling Technology, Danvers, MA, USA); anti- small mothers against decapentaplegic 2/3 (Smad)2/3, sc-133098, 1:300, Santa Cruz Biotechnology, Santa Cruz, CA, USA); anti-endothelium cell-specific receptor tyrosine kinase (Tie-2, sc-293414, 1:200, Santa Cruz Biotechnology, Santa Cruz, CA, USA); anti-angiopoietin 1 (Ang-1, bs-0800R, 1:500, Bioss Antibodies Inc., Woburn, MA, USA); anti-angiopoietin 2 (Ang-2, sc-74402, 1:100, Santa Cruz Biotechnology, Santa Cruz, CA, USA); anti-vascular endothelial growth factor (VEGF, sc-53462, 1:300, Santa Cruz Biotechnology, Santa Cruz, CA, USA); anti-vascular endothelial growth factor receptor 2 (VEGFR2, cod. 2472S, 1:500, Cell Signaling Technology, Danvers, MA, USA); anti-vitamin D receptor (VDR, sc-13133, 1:500, Santa Cruz Biotechnology, Santa Cruz, CA, USA); or anti-angiotensin II receptor type-1 (AT1, sc-515884, 1:200, Santa Cruz Biotechnology, Santa Cruz, CA, USA) antibodies overnight at 4 °C. Membranes were incubated with anti-glyceraldehyde-3-phosphate dehydrogenase (GAPDH) monoclonal antibody (cod. 2118L, 1:1000; Sigma Chemical Company, St. Louis, MO, USA) overnight at 4 °C as a loading control. The membranes were then washed and incubated with horseradish peroxidase-conjugated goat anti-mouse (P0448, 1:5000; Dako Corporation, Glostrup, CPH, Denmark), anti-rabbit (P0447, 1:2000, 1:5000 or 1:10,000; Dako Corporation, Glostrup, CPH, Denmark), or anti-goat (sc-2768, 1:5000, Santa Cruz Biotechnology, Santa Cruz, CA, USA) antibodies for 1 h at room temperature. An imaging system (Kodak Gel Logic 2200, Austin, TX, USA) visualized the membrane-bound antibodies using enhanced chemiluminescence (ECL) reagents (Sigma-Aldrich, St. Louis, MO, USA). The band intensity was quantified by densitometry using ImageJ NIH image software 1.52A (http://www.nih.gov, accessed on 1 March 2021) and was reported in arbitrary units. As described in previous studies, protein quantitation was performed using the Bradford method [27].

### 2.9. Statistical Analysis

The Nonparametric Kruskal-Wallis test, followed by Dunn’s post-test, was used to analyze non-normally distributed data. The data of renal tissue TGF-β1 were transformed into log_e_ to obtain a normal distribution. An analysis of variance followed by the Newman-Keuls multiple comparisons test was used to analyze normally distributed data by the Kolmogorov-Smirnov test. Statistical analyses were performed using GraphPad Prism version 9.0 for Windows (GraphPad Software, San Diego, CA, USA). The data were expressed as means ±standard error of the mean (SEM). A *p* of < 0.05 was considered statistically significant.

## 3. Results

The ADR presented a significantly decreased body mass at 27 days of the experiment compared to the paricalcitol group. The ADR + paricalcitol also presented a significant reduction in body weight compared to the paricalcitol group (Table 1).

Differences in the 25 OHD, PTH, P_Ca_, and P_P_ levels in control, paricalcitol, ADR, and ADR + paricalcitol groups were not found (Table 1).

### 3.1. Paricalcitol Improved ADR-Induced Kidney Dysfunction

**Figure 2:**
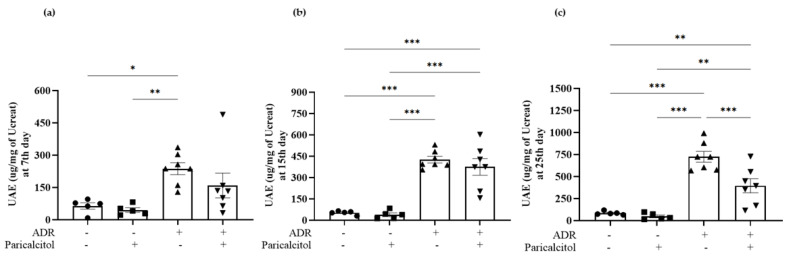
Urinary albumin excretion (UAE) at the 7th- day (**a**), 15th- day (**b**), and 25th- day (**c**) of the experiment. The data from the control (dots), paricalcitol (squares), ADR (up triangles), and ADR + paricalcitol (down triangles) groups at 27 days of paricalcitol treatment. *n* = 5–7 for each group. The data are expressed as the mean ±SEM. * *p* < 0.05; ** *p* < 0.01; *** *p* < 0.001.

Differences in urinary volume were not observed (Table 1). The UAE level on the 7th day after ADR injection was significantly increased in rats of the ADR group compared to the control (Figure 2a). On the 15th and 25th days after injection, this parameter was significantly higher in the ADR group than the control and paricalcitol groups (Figure 2b and 2c). The ADR + paricalcitol group had attenuation of this change on the 25th after the ADR injection, present significantly decreased compared to the ADR group (Figure 2c). Animals from the ADR group significantly reduced the GFR and increased FE_Na_ compared with the paricalcitol group. These alterations were less intense in the ADR + paricalcitol group than in the ADR group (Table 1).

### 3.2. Paricalcitol Attenuated ADR-Induced Renal Structure Injury

**Figure 3:**
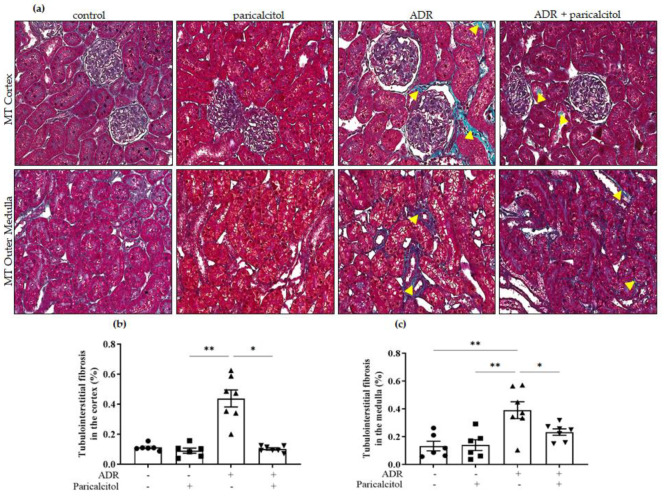
Representative Masson’s trichrome (MT) staining of kidney sections. (**a**). The top row represents the MT in the renal cortex, and the lower row represents the MT in the medullary compartment. The yellow arrowheads indicate the positive MT expression in tubulointerstitial compartments in the renal tissue. (**b**). Tubulointerstitial fibrosis in the renal cortex. (**c**). Tubulointerstitial fibrosis in the medullary compartment. Data from the control (dots), paricalcitol (squares), ADR (up triangles), and ADR + paricalcitol (down triangles) groups at 27 days of paricalcitol treatment. Scale bar = 20 μm, *n* = 6–7 for each group. Data are expressed as the mean ±SEM. * *p* < 0.05; ** *p* < 0.01.

The MT analysis showed tubulointerstitial fibrosis in the cortex, and the outer medulla was detected in the ADR group compared with the control and paricalcitol groups (Figure 3a–c). Additionally, the ADR + paricalcitol significantly attenuated the tubulointerstitial fibrosis in the cortex and the medulla compared with the ADR group (Figure 3a–c).

### 3.3. Paricalcitol Treatment Improved Endothelium Structure and Function

**Figure 4:**
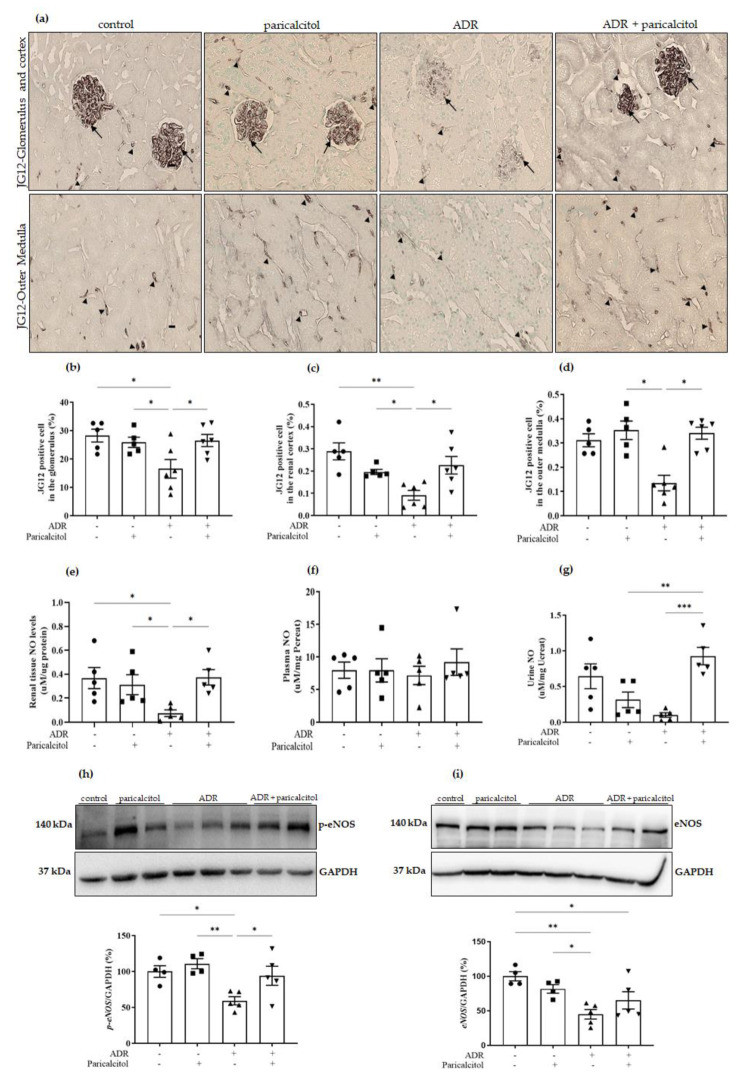
(**a**). The immunolocalization of JG12 in all renal compartments. The arrows indicate the JG12-positive expression in the glomerulus. The arrowheads indicate the JG12-positive expression in the tubulointerstitial compartments. (**b**). Percentual of JG12-positive cells in the glomerulus. (**c**). Percentual of JG12-positive cells in the renal cortex. (**d**). Percentual of JG12-positive cells in the outer medulla. Immunohistochemical data are expressed as the mean ± SEM. Scale bar = 20 μm, *n* = 5–6 for each group. (**e**). Renal tissue NO levels. (**f**). Plasma NO. (**g**). Urine NO, *n* = 5–6 for each group. Densitometric ratios among (**h**) p-eNOS or (**i**) eNOS and GAPDH were calculated, and data were expressed compared with the control group. The control value was designated as 100%. Data are expressed as mean ± SEM. *n* = 4–5 for each group. Data from the control (dots), paricalcitol (squares), ADR (up triangles), and ADR + paricalcitol (down triangles) groups. * *p* < 0.05; ** *p* < 0.01; *** *p* < 0.001. *Pcreat*. Plasma creatinine; *Ucreat*. Urine creatinine.

The expression of JG12, an EC marker, was reduced in the renal glomerulus and the tubulointerstitial compartments of the renal cortex and outer medulla in the ADR group compared to the control groups. In the animals treated with paricalcitol, there was attenuation in these alterations compared to the ADR group (Figure 4a–d).

The quantification of renal tissue NO levels showed a significantly decreased in the ADR group compared to the control and paricalcitol group, which were recovered in the ADR + paricalcitol group (Figure 4e). Urine NO levels decreased in the ADR group compared to the control groups. In contrast, these levels were higher in the ADR + paricalcitol group than in the paricalcitol and ADR groups (Figure 4g). In NO plasma levels, we did not find changes between the groups (Figure 4f). Densitometric ratios of *p-eNOS* (Figure 4h) and *eNOS* (Figure 4i) showed significantly decreased expression in the ADR group compared to the control and paricalcitol groups. Only *p-eNOS* was significantly increased in the ADR + paricalcitol group compared with the ADR group (Figure 4h).

### 3.4. Paricalcitol Treatment Attenuated the Expression of Mesenchymal Markers

**Figure 5:**
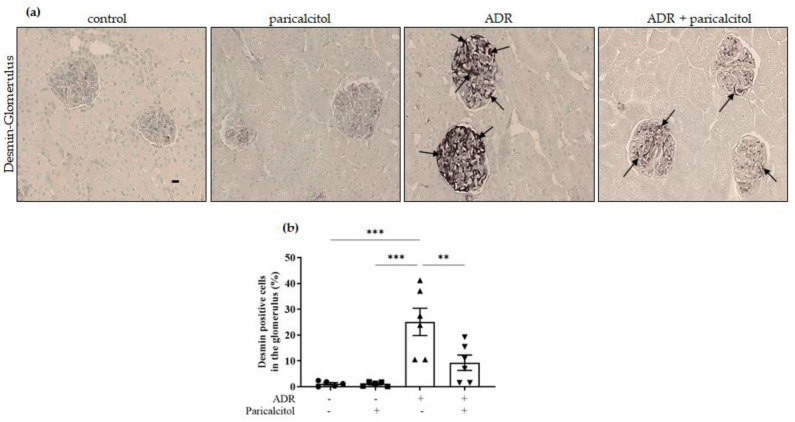
(**a**). The immunolocalization of desmin in the glomerulus. The arrows indicate the desmin-positive expression in the glomerulus. (**b**). Percentual of desmin-positive in the glomerulus from the control (dots), paricalcitol (squares), ADR (up triangles), and ADR + paricalcitol (down triangles) groups. Immunohistochemical data are expressed as the mean ± SEM. Scale bar = 20 μm, *n* = 5–6 for each group. ** *p* < 0.01; *** *p* < 0.001.

Desmin expression, a podocyte cell dedifferentiation marker in the glomerulus, was significantly increased in the groups that received ADR compared to the control groups. This expression was attenuated by paricalcitol treatment in the ADR + paricalcitol (Figure 5a,b).

**Figure 6:**
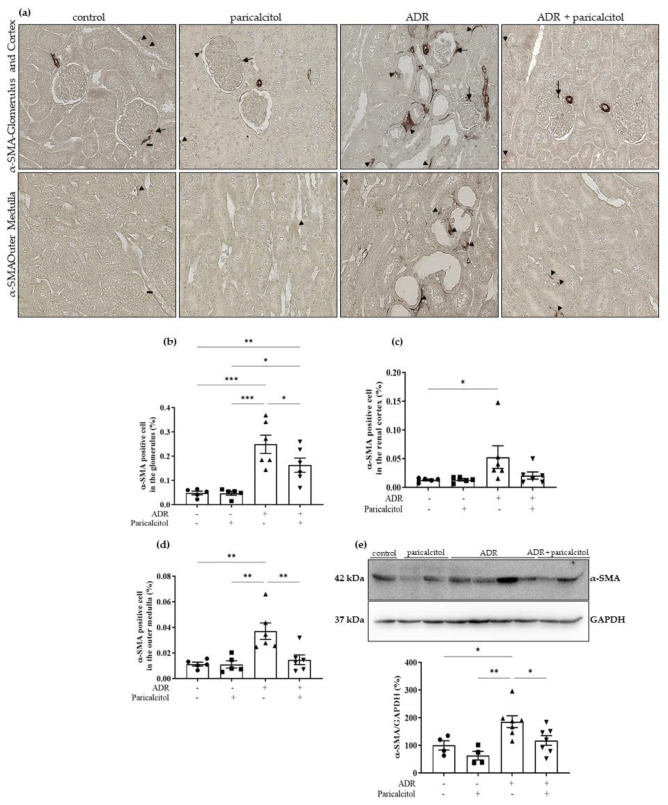
(**a**). The immunolocalization of α-SMA in all renal compartments. The arrows indicate the α-SMA-positive expression in the glomerulus. The arrowheads indicate the α-SMA-positive expression in the tubulointerstitial compartments. (**b**). Percentual of α-SMA-positive cells in the glomerulus. (**c**). Percentual of α-SMA-positive cells in the renal cortex. (**d**). Percentual of α-SMA-positive cells in the outer medulla. Immunohistochemical data are expressed as the mean ±SEM. Scale bar = 20 μm, *n* = 5–6. (**e**). Densitometric ratios among α-SMA and GAPDH were calculated, and data were expressed compared with the control group. The control value was designated as 100%. Data are expressed as mean ± SEM. *n* = 4–7 for each group. Data from the control (dots), paricalcitol (squares), ADR (up triangles), and ADR + paricalcitol (down triangles) groups. * *p* < 0.05; ** *p* < 0.01; *** *p* < 0.001.

The significantly increased expression of α-SMA was observed in the glomerulus, renal cortex, and outer medulla of animals from the ADR group compared to the control and paricalcitol groups. The ADR + paricalcitol group showed a decrease in this expression in the glomerulus and outer medulla. However, this alteration was not found in the renal cortex (Figure 6a–d). The densitometric analysis of α-SMA expression performed by Western blot studies showed increased expression in the renal tissue of animals from the ADR group compared to the control groups. In contrast, the ADR + paricalcitol group presented a significantly decreased expression of this protein (Figure 6e).

**Figure 7:**
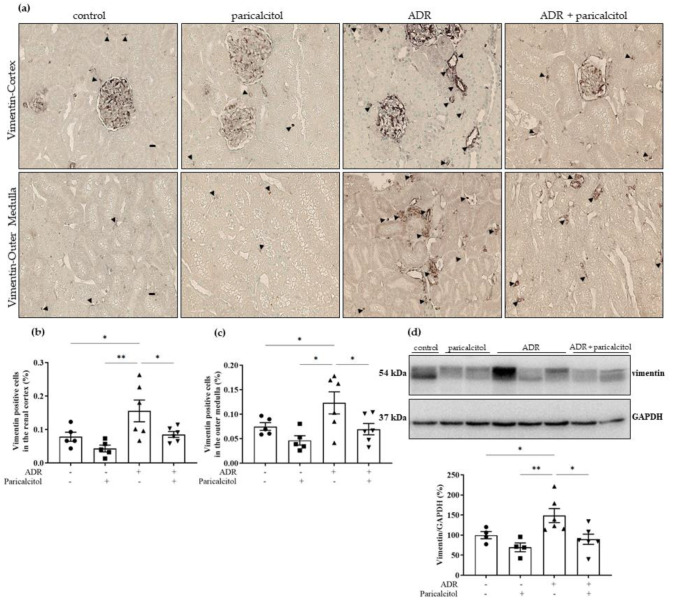
(**a**). The immunolocalization of vimentin in all renal compartments. The arrowheads indicate the vimentin-positive expression in the tubulointerstitial compartments. (**b**). Percentual of vimentin-positive cells in the renal cortex (**c**). Percentual of vimentin-positive cells in the outer medulla. Immunohistochemical data are expressed as the mean ±SEM. Scale bar = 20 μm, *n* = 5–6. (**d**). Densitometric ratios among vimentin and GAPDH were calculated, and data were expressed compared with the control group. The control value was designated as 100%. Data are expressed as mean ± SEM. *n* = 4–6 for each group. Data from the control (dots), paricalcitol (squares), ADR (up triangles), and ADR + paricalcitol (down triangles) groups. * *p* < 0.05; ** *p* < 0.01.

The expression of vimentin was significantly increased in the renal cortex and outer medulla of the animals from the ADR group compared to the control and paricalcitol groups. The ADR + paricalcitol group showed a decrease in this expression in these compartments related to the ADR group (Figure 7a–c). The expression of vimentin performed by Western blot studies showed significantly increased expression in the renal tissue of animals from the ADR group compared to the control and paricalcitol groups. The treatment with paricalcitol inhibited this increase in the ADR + paricalcitol (Figure 7d).

**Figure 8:**
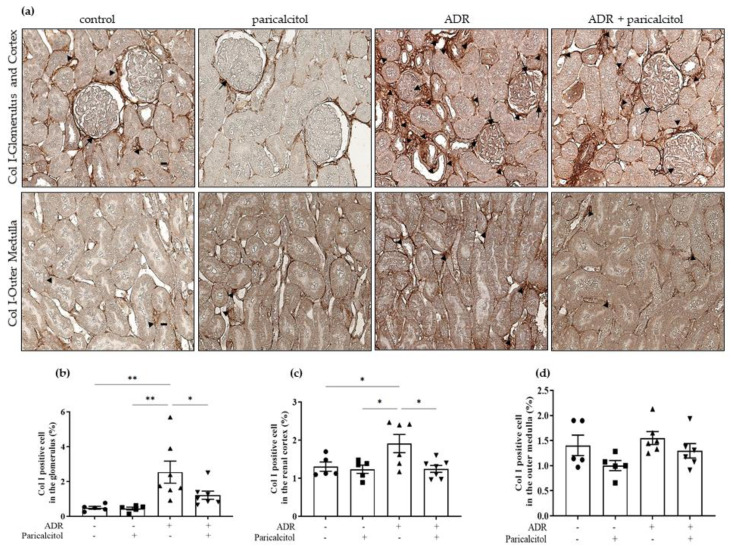
(**a**). The immunolocalization of Col I in all renal compartments. The arrows indicate the Col I-positive expression in the glomerulus. The arrowheads indicate the Col I-positive expression in the tubulointerstitial compartments. (**b**). Percentual of Col I-positive in the glomerulus. (**c**). Percentual of Col I-positive in the renal cortex. (**d**). Percentual of Col I-positive in the outer medulla. Immunohistochemical data are expressed as the mean ± SEM. Scale bar = 20 μm. *n* = 5–7 for each group. Data from the control (dots), paricalcitol (squares), ADR (up triangles), and ADR + paricalcitol (down triangles) groups. * *p* < 0.05; ** *p* < 0.01.

Finally, we found an increase in the Col I expression in the glomerulus and renal cortex of the ADR animals compared with the control group. The ADR + paricalcitol group showed a significantly decreased in this expression observed in the renal cortex of the rats from the ADR groups (Figure 8a–c). However, in the outer medulla, the rats from the different groups did not present a change in the Col I expression (Figure 8d).

### 3.5. Paricalcitol Modulated TGF-β1 through the Smads Pathway

**Figure 9:**
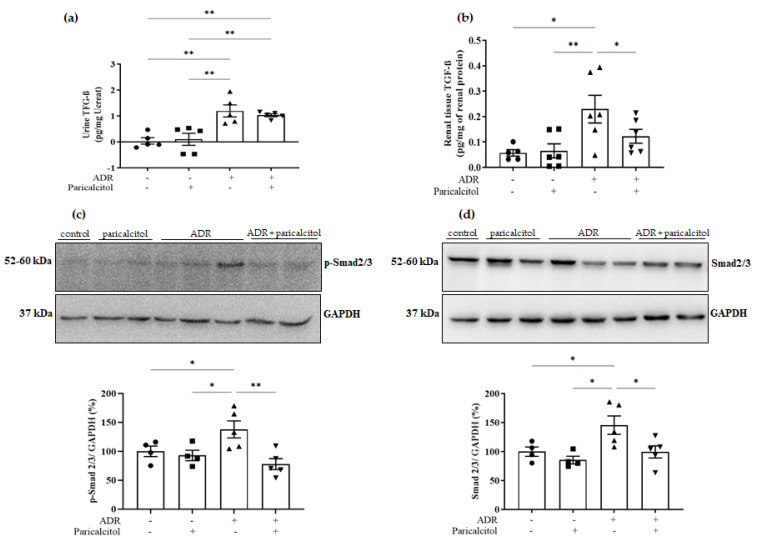
(**a**). Urine TGF-β1 levels. (**b**). Renal tissue TGF-β1 levels, *n* = 5–7 for each group. (**c**). Densitometric ratios among p-Smad2/3. (**d**). Densitometric ratios among Smad2/3. Densitometric ratios among these markers and GAPDH were calculated, and data were expressed compared with the control group. The control value was designated as 100%. Data are expressed as mean ± SEM. *n* = 4–5 for each group. Data from the control (dots), paricalcitol (squares), ADR (up triangles), and ADR + paricalcitol (down triangles) groups. * *p* < 0.05; ** *p* < 0.01.

Urine TGF-β1 was increased in the groups that received adriamycin (ADR and ADR + paricalcitol) compared with the control and paricalcitol groups. The paricalcitol treatment did not change this alteration. In the renal tissue TGF-β1 levels, the ADR group presented upregulation compared to the control and paricalcitol groups. In contrast, this alteration was significantly attenuated in the ADR + paricalcitol group. The same occurred with p-Smad2/3 and total Smad2/3, which were increased in the ADR group compared to control and paricalcitol groups. The p-Smad2/3 and total Smad2/3 expression were reduced in the animals from ADR + paricalcitol compared to the ADR group (Figure 9a–d).

### 3.6. Paricalcitol Treatment Attenuated Imbalance in Pro- and Anti-Angiogenic Factors

**Figure 10:**
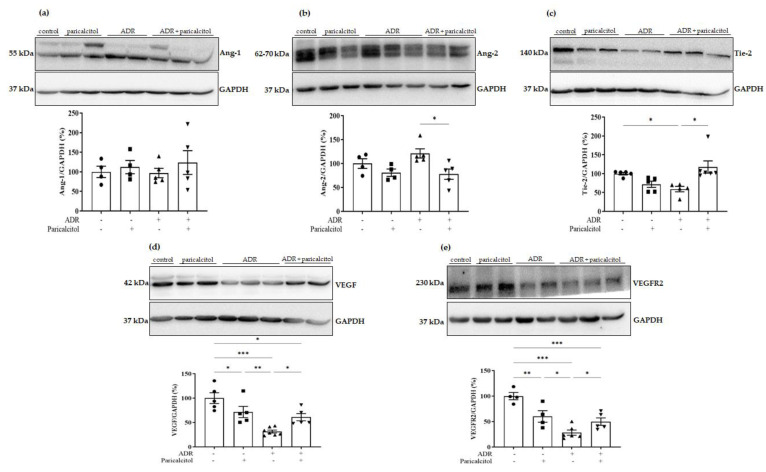
Pro- and anti-angiogenic factors. (**a**). Densitometric ratios among Ang-1. (**b**). Densitometric ratios among Ang-2. (**c**). Densitometric ratios among Tie-2. (**d**). Densitometric ratios among VEGF and (**e**). Densitometric ratios among VEGFR2. Densitometric ratios among these markers and GAPDH were calculated, and data were expressed compared with the control group. The control value was designated as 100%. Data are expressed as mean ± SEM. *n* = 5–7 for each group. Data from the control (dots), paricalcitol (squares), ADR (up triangles), and ADR + paricalcitol (down triangles) groups. * *p* < 0.05; ** *p* < 0.01; *** *p* < 0.001.

Differences in Ang-1 expression were not observed (Figure 10a). On the other hand, Ang-2 showed a tendency to increase in the ADR group, which was significantly reduced with paricalcitol treatment (Figure 10b). The expression of Tie-2 was decreased in the ADR group compared to the control group, and treatment with paricalcitol significantly increased this parameter compared to the ADR group (Figure 10c).

VEGF and their endothelial receptor, VEGFR2, decreased expressions in the paricalcitol group compared to the control. The ADR group also presented a significant reduction in this expression compared to the control and paricalcitol groups. In the ADR + paricalcitol group, the animals showed an increase in VEGF and VEGFR2 expression compared with the control and ADR groups (Figure 10c,d).

### 3.7. Paricalcitol Treatment Modulated AT1 and VDR Expression

**Figure 11:**
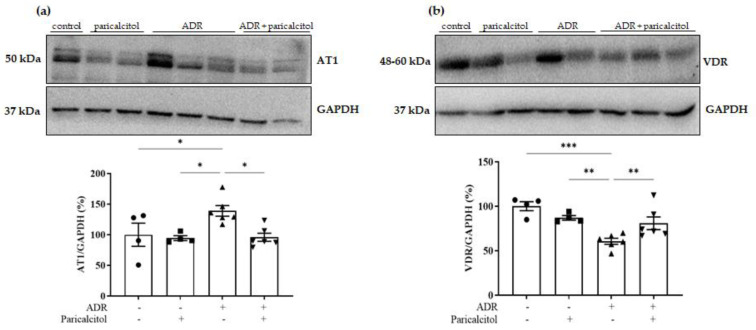
(**a**). Densitometric ratios among AT1. (**b**). Densitometric ratios among VDR. Densitometric ratios among these markers and GAPDH were calculated, and data were expressed compared with the control group. The control value was designated as 100%. Data are expressed as mean ± SEM. *n* = 4–6 for each group. Data from the control (dots), paricalcitol (squares), ADR (up triangles), and ADR + paricalcitol (down triangles) groups. * *p* < 0.05; ** *p* < 0.01; *** *p* < 0.001.

The expression of the AT1 receptor was increased in the ADR group compared to the control and paricalcitol groups. The ADR + paricalcitol group presented a significant decrease in this expression (*p* < 0.05) (Figure 11a).

We then investigated whether paricalcitol treatment could modulate VDR expression by itself. VDR expression was remarkably reduced in the ADR group compared to the control and paricalcitol groups. This expression in the ADR + paricalcitol group significantly increased compared to the ADR group (Figure 11b).

## 4. Discussion

The present work showed that paricalcitol treatment improved renal function and structure through antiproteinuric effects and glomerular and tubular protection. The activation of the VDR promoted the reduction in the renal microvasculature disturbances by attenuating the TGF-β1/Smad2/3-dependent and downregulation of Ang-2 and AT1 expression, regulating the Angs/Tie-2 and VEGF/VEGFR2 axes, which presents disturbances in ADR-induced nephropathy in rats. Our results showed a protective role of paricalcitol in the renal microvasculature that could be used as a target for treating the beginning of CKD.

Proteinuria is the most common clinical manifestation of glomerular diseases and is directly linked to kidney injury progression [29,30]. Several clinical and experimental studies have shown the participation of proteinuria in the tubulointerstitial lesion observed in glomerulopathies [31,32]. The results showed that renal function was reduced in the animals from the ADR group. A decrease in the GFR and an increase in FE_Na_ and UAE were observed. Histopathological analysis showed tubulointerstitial lesions and fibrosis in response to increased protein glomerular permeability, similar to other studies [33]. The pleiotropic action of vit. D has been recently studied [34]. Paricalcitol selectively activates VDR [20] by binding and interacting with VDR/vit. D response element (VDRE) and is essential in preventing morphological changes of podocytes in the SN model [35]. 1,25-dihydroxyvitamin D3 (1,25-D3), the active form of vit. D3 reduced a transendothelial albumin passage induced by ADR in vitro and in vivo via the interaction with VDR-heparanase promoter in podocytes [36]. Garsen et al. have suggested that crosstalk could exist between glomerular ECs and podocytes to induce proteinuria and vit. D can modulate this effect in these cells, mainly in podocytes with more VDR [36]. Paricalcitol reduced ADR-induced changes in renal function and structure, independent of 25 OHD and PTH levels. This data may reinforce that paricalcitol improves non-SHPT in CKD by acting directly on albuminuria parameters and reducing side effects without modifying the calcium-phosphorus product.

Previously, our laboratory showed that calcitriol treatment protects endothelial maintenance induced by RAS dysregulation during kidney development [27]. Our results showed an epithelial/EC loss in their phenotype, supported by data demonstrating a significant decrease in JG12, an endothelium marker, and an increased mesenchymal cell phenotype, positive for desmin, α-SMA, vimentin, and Col I in the renal interstitium and glomeruli, as seen previously in other works [3,20,37]. Paricalcitol treatment could reduce α-SMA and vimentin expression in the renal tissue in ADR animals. These data suggest that the alterations provoked by ADR can be modulated by paricalcitol, at least partly, due to its properties in cell differentiation and endothelium maintenance.

TGF-β1/Smad is the key to fibrogenic pathways in ADR-induced nephropathy [38], which is directly associated with ESRD [39] with Col I and fibronectin expression in renal tissue [1,40]. Smad family molecules are involved in the downstream of TGF-β1 after its phosphorylation [1], leading to the loss of endothelium function and fibroblastic phenotype [41]. Our data showed that the TGF-β1/p-Smad2/3 signaling was upregulated in the animals from the ADR group in renal tissue. Smad2 and Smad3 are mediators of extracellular matrix (ECM) production and activation of myofibroblasts via TGF-β1 [42] by binding to the promoter of collagens and inducing its production in CKD models and α-SMA induction in proximal-tubule epithelial human cells [43]. Tsai et al. have shown that the vit. D decreases p-Smad2/Smad2 expression in the human umbilical vein endothelial cells (HUVECs) and, in ADR-induced cardiotoxicity, inhibits tissue fibrosis [44]. The TGF-β1/p-Smad pathway is associated with the AT1 receptor. AT1 blockade in mitral valve EC contributes to the reduction in EndoMT by the decrease in TGF-β1/Smad activation [19]. Our study showed a reduction in the AT1 expression by paricalcitol treatment, indicating less vasoconstriction reflected in GFR and endothelium function. Paricalcitol-VDR activation reduces the RAS activation, decreasing renal *RENIN* and *AT1R* gene expression in CKD [15]. Thus, Vit. D protects microvasculature and tissue perfusion, improving capillary blood flow [45] and decreasing RAS activation and the expression of its components directly associated with the TGF-β1/Smad pathway in kidney fibrosis. The urinary TGF-β1 is indicative of CKD and was increased in ADR animals. However, paricalcitol treatment did not attenuate this parameter.

We also observed increased expression of p-eNOS/NO tissue levels in animals treated with paricalcitol. This improvement is due to the increase in either expression or activity of eNOS, resulting in more efficient NO production [46,47]. In the ADR model, there are alterations in the NO pathways, probably due to endotheliotoxicity, inhibiting and uncoupling eNOS, preventing its phosphorylation and NO availability [13,48]. Paricalcitol is involved in regulatory mechanisms in NO pathways preventing reactive oxygen species (ROS) production and free radicals [14,17]. Vit. D/VDR stimulates the NOS3 gene, resulting in higher NO production, which improves vascular function [49]. In this case, eNOS could phosphorylate VEGF and vice-versa, protecting podocyte loss, glomerular and peritubular vascular rarefaction, renal fibrosis [50], and inhibiting the expression of TGFβ-Smad3 [51]. Our work evidenced the synergistic effect of VDR activation and pro-angiogenic factors. These effects can cause neovascularization and maintenance of the endothelium by increasing VEGF levels in tissue [52,53], probably by acting on the proliferation and differentiation of cells [10,27]. The VEGF has pro- and anti-fibrotic effects at different times of kidney health and diseases to regulate angiogenesis and also plays a role in the progression of renal fibrosis [51]. We have seen a reduction in the VEGF/VEGFR2 expression in animals from the paricalcitol group, which reinforces the evidence that there is a certain duality of the effects of VEGF that exacerbates or inhibits the process of renal fibrosis. In our study, paricalcitol regulates VEGF to act in different ways.

Vit. D deficiency increases Ang-2 levels in renal tissue associated with lower Ang-1 and Tie-2 receptor [11]. Ang-1 acts as an angiogenic factor stimulating the proliferation and maturation of EC in angiogenesis, fibrosis, and inflammation [11,54] associated with attenuation of myofibroblast activation, ECM accumulation, and peritubular capillary growth observed in UUO model [9,55]. Ang-2 is associated with increased albuminuria and decreased GFR in diabetes mellitus type 2 and advanced CKD [24,25,56]. The higher Ang-1: Ang-2 ratio has been associated with 72% less CKD progression and 82% lower mortality risk in acute kidney injury (AKI) [57]. Paricalcitol treatment attenuated alterations in Ang-2 expression, which reflected in the downregulation of the Ang-2/Tie-2 pathway, reducing the albuminuria, alterations in the GFR, and vascular rarefaction in animals received paricalcitol treatment. Furthermore, Tie-2 and VEGFR2 are specific endothelial receptors that support these data together with the recovery of JG12 and VDR receptor expression in renal cells. This demonstrates that vit. D in its active and circulating form can increase the VDR in the face of the onset of an ADR-induced kidney injury. Our results reinforced the essential role of the renal medulla compartment in kidney development [27] and adulthood kidney function efficiency. Further studies through in vitro experiments using primary endothelial cells of the kidney and angiogenesis assays should be performed to evaluate this process better in the microvasculature of both the renal cortex and the medulla. Despite this, with the limitations involving experimental models, the present study adds to the literature concerning the participation of endothelial cells and their markers in early CKD.

## 5. Conclusions

In conclusion, paricalcitol attenuates microvasculature alterations in ADR-induced kidney injury in rats. Vit. D had antiproteinuric effects and cellular differentiation by inhibiting the TGF-β1/Smad2/3 pathway, regulating the Angs/Tie-2, VEGF/VEGFR2, and AT1 axes through recovery endothelial structure and function, and VDR expression in renal tissue. Our data also emphasized the importance of the renal medulla and peritubular capillaries in maintaining renal function and structure. The results suggest that paricalcitol could potentially be therapeutic in preventing renal fibrosis by targeting the endotheliotoxicity caused by early CKD.

## Figures and Tables

**Table 1:** Body weight, 25-hydroxyvitamin D, parathyroid hormone, plasma calcium concentration, plasma phosphorus concentration, 24-h urine volume, glomerular filtration rate, and fractional excretion of sodium at the end of the experiment in the control, paricalcitol, ADR, and ADR + paricalcitol groups.

	Control	Paricalcitol	ADR	ADR + Paricalcitol
Body Weight (g)	402 ± 21.70	451 ± 17.30	392 ± 11.90 #	354 ± 12.70 ###
25 OHD (ng/mL)	29 ± 3.28	34 ± 1.96	28 ± 2.19	31 ± 1.39
PTH (pg/mg)	177 ± 22.40	180 ± 36.40	288 ± 29.50	270 ± 35.70
P_Ca_ (mg/dL)	9.1 ± 0.27	9.4 ± 0.5	9.1 ± 0.33	9.5 ± 0.24
P_p_ (mg/dL)	7.0 ± 0.46	6.7 ± 0.33	7.1 ± 0.23	7.6 ± 0.20
Urine volume (mL 100 g^−1^ 24 h^−1^)	5.1 ± 1.57	10.4 ± 2.24	8.3 ± 1.26	9.5 ± 1.03
GFR (ml min^−1^ 100 g^−1^)	0.3 ± 0.04	0.4 ± 0.03	0.2 ± 0.02 #	0.4 ± 0.01 $
FE_Na_ (%)	0.3 ± 0.02	0.2 ± 0.02	0.3 ± 0.03 #	0.2 ± 0.01 $

The data are expressed as mean ±SEM. *n* = 5–7 for each group. # *p* < 0.05, ### *p* < 0.001 vs. paricalcitol; $ *p* < 0.05 vs. ADR. 25OHD, 25-hydroxyvitamin D; PTH, parathyroid hormone; P_Ca_, plasma calcium concentration; P_P_, plasma phosphorus concentration; GFR, glomerular filtration rate; FE_Na_, fractional sodium excretion.

## Data Availability

Not applicable.
